# Advancements in Renal Imaging: A Comprehensive Systematic Review of PET Probes for Enhanced GFR and Renal Perfusion Assessment

**DOI:** 10.3390/diagnostics15243209

**Published:** 2025-12-15

**Authors:** Marwah Abdulrahman, Ahmed Saad Abdlkadir, Serin Moghrabi, Salem Alyazjeen, Soud Al-Qasem, Deya’ Aldeen Sulaiman Sweedat, Saad Ruzzeh, Dragi Stanimirović, Michael C. Kreissl, Hongcheng Shi, Mike Sathekge, Akram Al-Ibraheem

**Affiliations:** 1 Department of Nuclear Medicine, King Hussein Cancer Center (KHCC), Amman 11941, Jordan; 2 Institute of Nuclear Medicine and Thyroid Gland Disease, University Clinical Centre of the Republic of Srpska, 78000 Banja Luka, Bosnia and Herzegovina; 3 Division of Nuclear Medicine, Department of Radiology and Nuclear Medicine, University Hospital of Magdeburg, 39120 Magdeburg, Germany; 4 Department of Nuclear Medicine, Zhongshan Hospital, Fudan University, Shanghai 200032, China; 5 Department of Nuclear Medicine, University of Pretoria & Steve Biko Academic Hospital, Pretoria 0001, South Africa; 6 Nuclear Medicine Research Infrastructure (NuMeRI), Steve Biko Academic Hospital, Pretoria 0002, South Africa; 7 School of Medicine, University of Jordan, Amman 11942, Jordan

**Keywords:** positron emission tomography/computed tomography, PET/CT, EDTA, glomerular filtration rate, GFR, GFR tracers, renal scintigraphy

## Abstract

Glomerular filtration rate (GFR) is a key indicator of renal function. Traditional methods for GFR measurement have limitations including invasiveness, low spatial resolution, and lengthy protocols. Positron emission tomography (PET) radiotracers have emerged as promising tools for non-invasive, accurate, and dynamic renal function assessment. **Objectives:** This systematic literature review evaluates the clinical utility, and current evidence surrounding PET radiotracers used for GFR measurement in humans, emphasizing advances over conventional renal imaging modalities. **Methods:** A systematic literature search was conducted in PubMed, Web of Science, and Scopus, following the Preferred Reporting Items for Systematic Reviews and Meta-Analyses (PRISMA) guidelines, from database inception to November 2024. The search identified studies evaluating PET-based measurement of glomerular filtration rate (GFR) and renal perfusion. Inclusion criteria encompassed human studies using PET radiotracers (e.g., ^68^Ga, ^18^F) with comparisons to reference standards (estimated GFR or serum creatinine). Two authors independently screened titles/abstracts, extracted data, and assessed bias using Quality Assessment of Diagnostic Accuracy Studies tool (QUADAS-2). Exclusions included animal studies, reviews, and non-English articles. **Results:** Eleven studies met inclusion criteria, with ^68^Ga-EDTA showing the highest validation against reference standards such as ^51^Cr-EDTA plasma clearance, demonstrating strong correlation. PET imaging offered superior spatial–temporal resolution, enabling accurate split renal function assessment and quantitative analysis of both filtration and perfusion. ^68^Ga-somatostatin analogues exhibited moderate correlations between renal SUV and estimated GFR, with post-PRRT uptake changes indicating early nephrotoxicity. Among novel tracers, ^68^Ga-FAPI showed a strong inverse SUV–GFR relationship, reflecting renal fibrosis and suggesting potential as a chronic kidney disease (CKD) biomarker but requires further clinical validation. Limitations across studies include small sample sizes, retrospective designs, and variability in reference standards. **Conclusions:** PET radiotracers, particularly ^68^Ga-EDTA, represent a significant advancement for non-invasive, quantitative GFR measurement with improved precision and renal anatomical detail compared to traditional methods. Future prospective, large-scale human studies with standardized protocols are needed to establish these PET tracers as routine clinical tools in nephrology. Integration of hybrid PET/MRI and novel tracer development may further enhance renal diagnostic capabilities.

## 1. Introduction

Glomerular Filtration Rate (GFR) is considered the key indicator of renal function, representing the volume of plasma filtered by the glomeruli into the Bowman’s capsule, approximately 125 mL/min under normal physiological conditions, or about 20% of total renal plasma flow [1]. Renal perfusion can be used as a determinant of GFR, as adequate blood flow is required to maintain filtration pressure and ensure optimal solute clearance. Since alterations in renal perfusion often precede or accompany changes in GFR, both parameters must be assessed to achieve a comprehensive evaluation of renal function [2]. Precise GFR measurement is pivotal in various clinical settings, being used in the evaluation and monitoring of chronic kidney disease, directly impacts the dosing of numerous medications, including cytostatics, and for evaluating normal kidney function in potential kidney donors and renal transplant follow-up [3]. The previously predominant technique applied in clinical practice to achieve precise GFR measurements involves evaluating the plasma clearance of a substance that is solely excreted via glomerular filtration and devoid of tubular reabsorption or excretion. Inulin, an exogenous marker, is recognized as the gold standard for accurately estimating GFR, as it is freely filtered at the glomerulus and neither reabsorbed nor secreted by the renal tubules [4]. Alongside established nuclear medicine techniques, iohexol—a nonionic, low-osmolality contrast agent characterized by exclusive glomerular filtration without significant tubular secretion or reabsorption—has been established as a reference method for the precise determination of glomerular filtration rate (GFR) via plasma clearance. Its clearance kinetics permit quantification using comprehensive multi-point sampling or validated, simplified single-sample protocols. The utility of iohexol as a GFR tracer is further underscored by its favorable analytical profile, including cost-effectiveness, high in vitro stability, and minimal interlaboratory variability [5]. Despite its precision, its application is limited in clinical settings due to technical challenges and significant cost. In routine clinical practice, creatinine clearance is often used as a surrogate measure of GFR because creatinine is endogenously produced and easily measurable. However, creatinine is not a purely filtered substance, it undergoes partial tubular secretion, leading to an overestimation of true GFR, particularly in advanced renal impairment. Furthermore, serum creatinine levels are influenced by factors such as age, sex, muscle mass, diet, and certain medications, reducing its accuracy as a standalone indicator of renal function [6]. An alternative approach involves measuring GFR using chromium-51 ethylenediaminetetraacetic acid (^51^Cr-EDTA) or technetium-99m diethylenetriaminepentaacetic acid (^99^ᵐTc-DTPA). Both are small molecules that are cleared primarily, though not perfectly exclusively, by glomerular filtration. ^51^Cr-EDTA is considered the superior radiopharmaceutical marker for GFR due to its nearly identical pharmacokinetics to inulin, whereas the clearance of ^99^ᵐTc-DTPA can be slightly lower and more variable due to minor plasma protein binding [7].

An alternative approach involves measuring blood clearance using Cr-51-ethylenediaminetetraacetic acid (^51^Cr-EDTA) or Tc-99m diethylenetriaminepentaacetic acid (^99m^Tc-DTPA), both of which are exclusively cleared via glomerular filtration, allowing calculation of total renal function. ^51^Cr-EDTA is considered superior to ^99m^Tc-DTPA due to its pharmacokinetic similarity to the gold-standard inulin, whereas ^99m^Tc-DTPA can exhibit variable clearance [8]. Despite these advantages, blood clearance methods remain less widely applied because they are time-consuming and require multiple blood or urine samples [9]. The advent of positron emission tomography (PET) imaging, with its superior spatial and temporal resolution, increased sensitivity, absolute quantification, and three-dimensional imaging, has potentially enhanced renal parenchymal visualization compared to Single Photon Emission Computed Tomography (SPECT). This advancement allows for the generation of time-activity curves based solely on kidney uptake, while an automated standardized uptake value threshold can be applied to accurately delineate activity in the renal cortex and collecting system [10]. Advancements in PET technology, including time-of-flight capabilities and enhanced detectors, not only improve imaging efficacy but also allow for lower radioactive tracer doses, making PET particularly safe and advantageous for pediatric patients who require multiple sequential scans [10].

In PET renal imaging, both ^68^Ga and ^18^F-labelled pharmaceuticals are currently being explored for assessing renal function, with promise in preclinical and experimental studies. Among these, ^68^Ga-EDTA and 2-deoxy-2-18F-fluorosorbitol (^18^F-FDS) have garnered significant attention. Notably, most of these radiotracers have been extensively studied in small animal models, providing valuable insights into their pharmacokinetics, renal clearance, and potential for clinical translation [11,12,13]. No comprehensive systematic review of the accuracy of GFR measurement methods using PET radiotracers has been published. We aim to provide a comprehensive evaluation of PET radiotracers used in GFR measurement, summarizing the current evidence, assessing their accuracy and clinical utility, and identifying gaps that need to be addressed for broader clinical implementation. While numerous preclinical studies in animal models, particularly mice, this systematic review focuses solely on human studies, to ensure that the findings are directly applicable to clinical practice and avoid the potential limitations of translating preclinical data to human physiology.

## 2. Materials and Methods

This review was reported according to the Preferred Reporting Items for Systematic Reviews and Meta-Analyses (PRISMA) [14] guidelines and has not been registered with PROSPERO or any other registration platforms, and thus, does not have an assigned registration number. No ethical approval or informed consent was required (Table S1).

### 2.1. Search Strategy

A comprehensive literature search was performed to identify studies assessing PET-based GFR quantification, including all PET radiotracers, irrespective of comparison with gold-standard methods like inulin clearance. Three electronic databases (PubMed, Web of Science, and Scopus) were comprehensively and systematically searched from their inception to 1 November 2024, using the following search terms: (“Glomerular Filtration Rate” OR “GFR” OR “renal perfusion”) AND (“Positron Emission Tomography” OR “PET” OR “PET imaging”) AND (“radiotracer” OR “radiopharmaceutical” OR “tracer kinetics”). No restrictions were placed on study design, population, or comparator method, ensuring a comprehensive assessment of all relevant PET-based GFR studies. Additional references were identified through manual screening of citations from included studies and relevant reviews.

### 2.2. Eligibility Criteria

To ensure the inclusion of relevant studies, the following eligibility criteria were applied: only original research articles, clinical trials, case series, and case reports focusing on PET radiotracers for GFR measurement were included. Studies evaluating the use of PET-based radiotracers for assessing renal function in human subjects were considered. Reviews, editorials, conference abstracts, commentaries, animal studies on mice, and incomplete studies were excluded. Only studies published in English were considered to ensure the accuracy and consistency of data extraction.

### 2.3. Screening and Data Extraction

Two authors (M.A. and A.A-I.) have conducted the initial screening by evaluating the titles and abstracts of the records in Rayyan AI [15]. Following this, they independently performed a secondary screening by thoroughly reviewing the full text of the identified studies based on predetermined inclusion criteria. Subsequently, they independently extracted data from the included studies using a predesigned Microsoft Office Excel Professional Plus 2016 spreadsheet. The extracted data encompassed various aspects, such as study design, sample size, type of PET radiotracer used, method of GFR assessment, imaging protocols and clinical validation against standard GFR measurement techniques. The extracted data were reviewed and validated by the reviewers to ensure accuracy and completeness. Disagreements during the data extraction process were resolved through discussion, and a third reviewer (S.R.) was consulted when necessary. This meticulous approach aimed to ensure the reliability and consistency of the data extracted for the systematic review.

### 2.4. Risk of Bias and Quality Assessment

Next, the methodological quality of the included studies was independently assessed using the standardized Quality Assessment of Diagnostic Accuracy Studies (QUADAS-2) protocol [16], which assesses four key domains: patient selection, index test, reference standard, and flow and timing. Patient selection bias was evaluated based on study population characteristics, inclusion/exclusion criteria, and study design. The index test domain assessed the methodology and interpretation of PET radiotracer-based GFR measurements, including standardization of imaging protocols and tracer kinetics. The reference standard domain examined the reliability of the comparator GFR measurement, such as inulin clearance or creatinine clearance, and its potential influence on diagnostic accuracy. Flow and timing were analyzed to assess the completeness of the timing between PET imaging and reference standard tests and any data exclusions that could affect results.

## 3. Results

### 3.1. Study Selection

**Figure 1:**
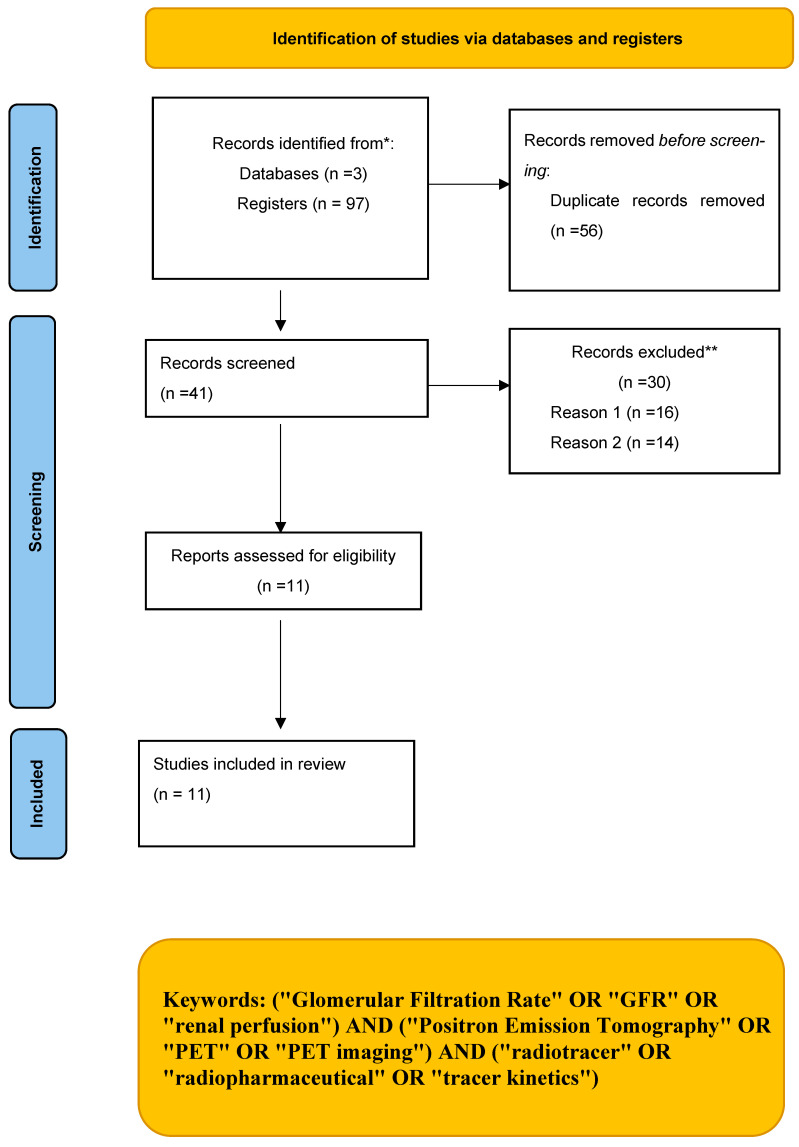
The selection process using the Preferred Reporting Items for Systematic Reviews and Meta-Analyses (PRISMA) flow diagram. * Databases: PubMed, Web of Science, and Scopus. ** Reason 1: Animal studies (*n* = 16); Reason 2: Review articles (*n* = 14).

The initial search identified 97 published articles, from which 56 duplicates were eliminated, leaving 41 records. Upon screening titles and abstracts, 16 studies on animals and 14 reviews were excluded. Finally, 11 studies—with 5 being prospective [17,18,19,20,21], one being a pilot study with humans [22], and the remaining being retrospective analyses [23,24,25,26,27]—were included in the review (Figure 1).

### 3.2. Characteristics of the Included Studies

Table 1 summarizes the included studies exploring the use PET radiotracers in GFR measurement. The studies were conducted in Germany, China, Australia, Canada, Japan, Thailand and Austria.

### 3.3. Risk of Bias and Quality Assessment

**Figure 2:**
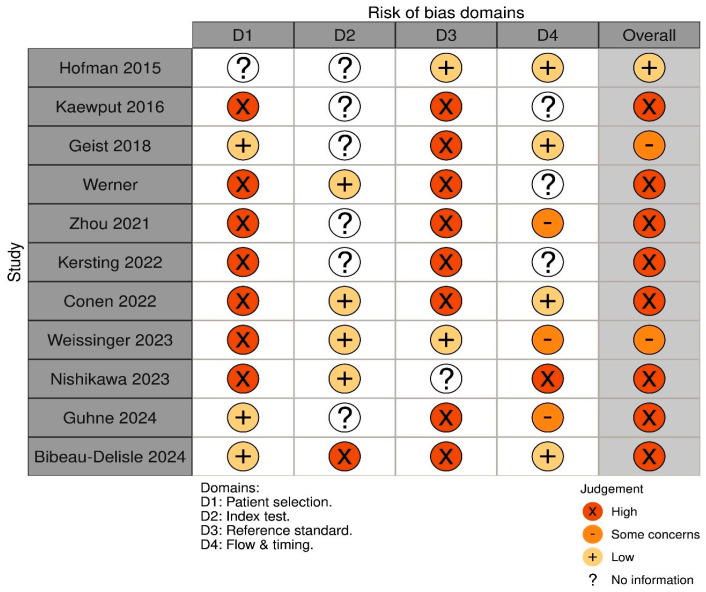
Risk of bias assessment of the included studies according to QUADAS 2 tool [17,18,19,20,21,22,23,24,25,26,27].

In this review, the risk of bias and quality assessment of the included 11 clinical studies using the QUADAS-2 tool revealed several trends regarding methodological quality across the included research, in which Hofman et al. (2015) [21] demonstrated low overall risk of bias, with robust reference standards and concurrent testing. Studies like Kaewput et al. (2016) [27], Kersting et al. (2022) [20], and Zhou et al. (2021) [19] faced high bias due to retrospective designs, small sample sizes, reliance on non-gold reference standards (e.g., serum creatinine), and methodological variability. Weissinger et al. (2023) [23] and Geist et al. (2018) [17] showed moderate bias, primarily from retrospective approaches, indirect reference standards, and partial-volume effects. Conen et al. (2022) [24], Gühne et al. (2024) [25] and Bibeau-Delisle et al. (2024) [26] had high applicability concerns due to reliance on surrogate markers (e.g., eGFR) and protocol heterogeneity. Nishikawa et al. (2023) [18] had a high bias in patient selection but low applicability concerns. Key recurring limitations included retrospective designs, non-standardized protocols, and underpowered cohorts, emphasizing the need for larger prospective studies using gold-standard comparators to validate PET-based GFR methods (Figure 2).

## 4. Systematic Review

### 4.1. ^68^Ga-Labelled Radiotracers for Renal Functional Imaging

#### 4.1.1. ^68^Ga-EDTA

**Figure 3:**
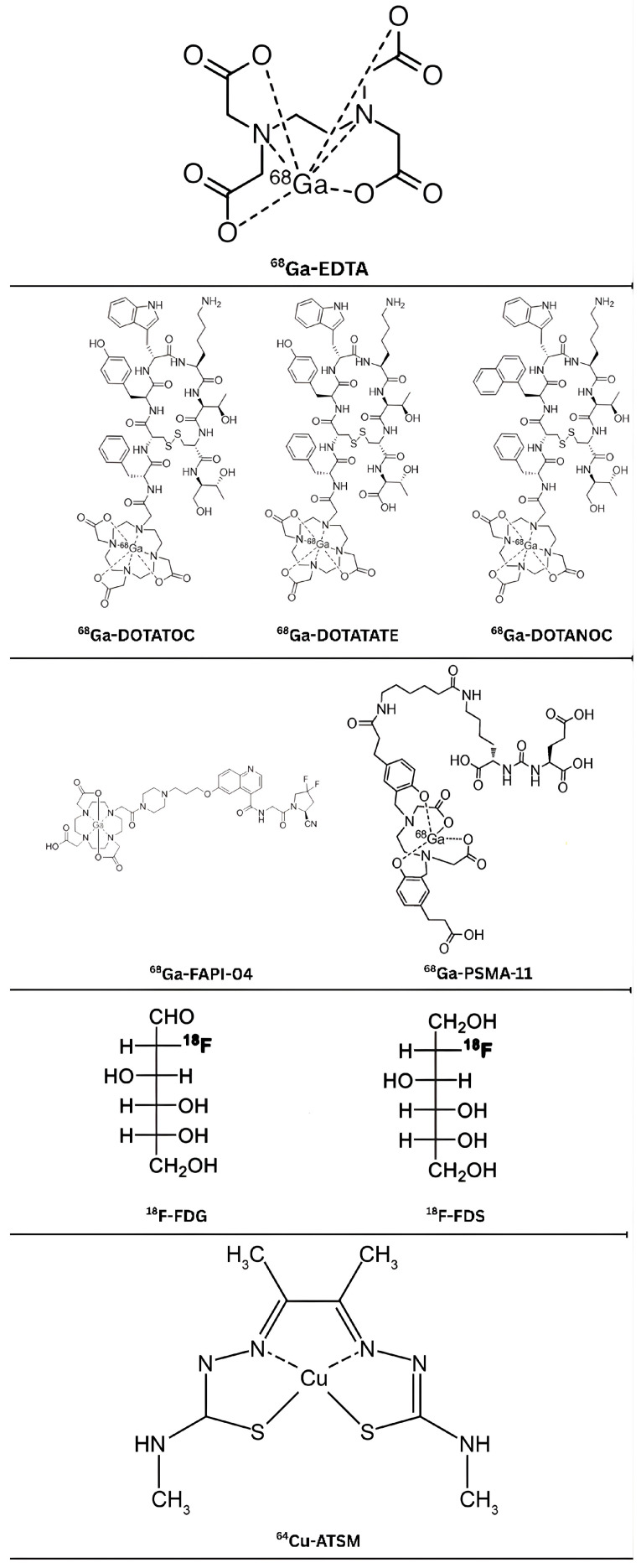
Chemical structure of PET radiopharmaceuticals used in GFR. Original image created by the author.

^68^Ga-EDTA, a stable metal chelate, binds strongly to Ga^3+^ with a formation constant (log K_1_ = 21.7), ensuring high thermodynamic stability. Its low protein binding (2.8% ± 0.6%) and exclusive clearance via glomerular filtration make it ideal for renal imaging. Rapid blood clearance (biological half-life ~1.47 hours) and urinary excretion (34% activity excreted) underpin its pharmacokinetics, providing high target-to-background contrast in PET imaging [10]. Safety is underscored by a low effective radiation dose (0.04 mSv/MBq for adults), comparable to ^99m^Tc-DTPA, and minimal EDTA content, 1/500,000th of therapeutic doses used for lead poisoning. No adverse reactions or nephrotoxicity are reported, even in severe renal impairment, making it a safe option across patient populations [10] (Figure 3).

Hofman et al. [21] were the first to introduce ^68^Ga-EDTA PET/CT as a potential alternative for GFR measurement for its ability to provide both imaging and accurate quantification of renal clearance. Their study evaluated and compared GFR estimation using simultaneous injection of ^68^Ga-EDTA and ^51^Cr-EDTA in 25 patients, with PET/CT-derived GFR. Plasma-derived GFR ranged from 10 to 220 mL/min (mean: 85 ± 48 mL/min), with patients categorized into severe (n = 2), moderate (n = 8), and mild (n = 8) renal impairment, while 13 had normal function. Patients were primarily assessed for neuroendocrine tumors (n = 17) or renal cell carcinoma (n = 14), with the latter undergoing split renal function evaluation before surgery or stereotactic radiotherapy. A near-perfect correlation (PCC = 0.94) was observed between ^68^Ga-EDTA and ^51^Cr-EDTA plasma sampling. However, ^68^Ga-EDTA underestimated GFR at values exceeding 150 mL/min. PET/CT acquisition included dynamic imaging (0–10 min) and repeated kidney-to-bladder imaging (11–20 min), with plasma sampling at 2, 3, and 4 h. A Bland–Altman analysis demonstrated good agreement between ^68^Ga-EDTA and ^51^Cr-EDTA methods. The study also highlighted the potential of ^68^Ga-EDTA PET in providing additional insights into split renal function assessment using ^99m^Tc-Dimercaptosuccinic Acid (DMSA) during the initial phase of renal parenchymal transit [21].

#### 4.1.2. ^68^Ga-DOTANOC, ^68^Ga-ha DOTATATE

In 2016, a study by Kaewput and Vinjamuri [27] investigated the correlation between renal uptake of ^68^Ga-DOTANOC PET/CT and eGFR before and after peptide receptor radionuclide therapy (PRRT) in patients with metastatic neuroendocrine tumors (NETs). Given the nephrotoxicity risks associated with PRRT, renal function assessment is crucial. The study retrospectively analyzed ^68^Ga-DOTANOC PET/CT scans from 32 patients undergoing ^90^Y-DOTATATE therapy. Renal uptake was quantified as the mean standardized uptake value (SUVmean) after background subtraction, and eGFR was calculated using standard software. The findings showed only fair agreement between SUVmean and eGFR before PRRT (r = 0.33) and poor agreement after PRRT (r = 0.16). There was a statistically significant decline in eGFR following PRRT (mean difference = 4.41 ± 9.24 mL/min/1.73 m^2^, *p* = 0.01). Interestingly, renal uptake on ^68^Ga-DOTANOC PET/CT increased significantly after PRRT (SUVmean change = −1.25 ± 3.17, *p* = 0.03), suggesting a possible early indicator of renal dysfunction. Although renal quantitative analysis using ^68^Ga-DOTANOC PET/CT did not strongly correlate with eGFR, the observed increase in uptake post-PRRT raises the possibility of using this imaging biomarker for early renal toxicity detection [27].

**Figure 4:**
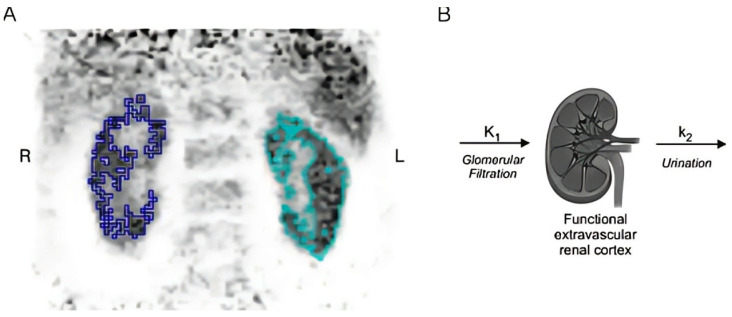
Example of functional renal cortex segmentation and tracer kinetic modeling in dynamic ^68^Ga-DOTA PET imaging. (**A**) Frontal view of the first PET frame illustrating the segmentation of the functional renal cortex used to derive time–activity curves. (**B**) Schematic representation of the single-compartment tracer kinetic model applied to estimate glomerular filtration rate (GFR). The arterial input function was extracted from the abdominal aorta, and the model was fitted to dynamic PET data over 30 min post-injection to obtain GFR values, with an additional analysis using reduced 15 min data sets to evaluate the feasibility of shorter acquisitions. This figure is adapted from reference [20], available under a Creative Commons Attribution (CC BY 4.0) license.

Kersting et al. (2022) [20] investigated the utility of dynamic renal ^68^Ga-DOTA PET/CT for non-invasive GFR estimation in 12 patients undergoing radionuclide therapy, comparing it to renal scintigraphy ^99m^Tc-DTPA or ^99m^Tc-MAG3 and serum creatinine-derived GFR (CKD-EPI formula). Using single-compartment kinetic modeling, GFR was derived from 30 min (GFRPET-30) and abbreviated 15 min (GFRPET-15) PET acquisitions, with adjustments to mitigate urinary spill-over effects. Dynamic PET data were acquired over 30 min using a silicon-photomultiplier-based PET/CT system, with subsequent reconstruction and analysis involving single-compartmental kinetic modeling to calculate GFR. The arterial input function was derived from the abdominal aorta, and renal cortical time–activity curves were generated by segmenting renal cortices, employing varying frame durations to capture both the bolus and clearance phases. Visual interpretation of PET images, which offered higher spatial and temporal resolution compared to scintigraphy, aligned with scintigraphy findings, detecting urinary obstructions and revealing additional anatomical details like renal cysts and aortic aneurysms. PET-derived GFR, both from 30 min (GFRPET-30) and 15 min (GFRPET-15) data, showed good correlation with serum creatinine-derived GFR, particularly in patients with unobstructed urinary flow, where correlations were excellent, as confirmed by intraclass correlation coefficients and Bland–Altman analysis. Notably, 15 min PET scans achieved near-identical accuracy to 30 min protocols (ICC 0.96–0.99), supporting reduced imaging durations. While the study highlights PET’s potential for simultaneous anatomical and functional renal assessment without blood sampling, limitations include its small cohort, retrospective design, and reliance on CKD-EPI rather than gold-standard inulin clearance. These findings position ^68^Ga-DOTA PET/CT as a viable alternative to scintigraphy, though larger prospective validations with direct GFR methods are critical to confirm clinical applicability [20] (Figure 4).

**Figure 5:**
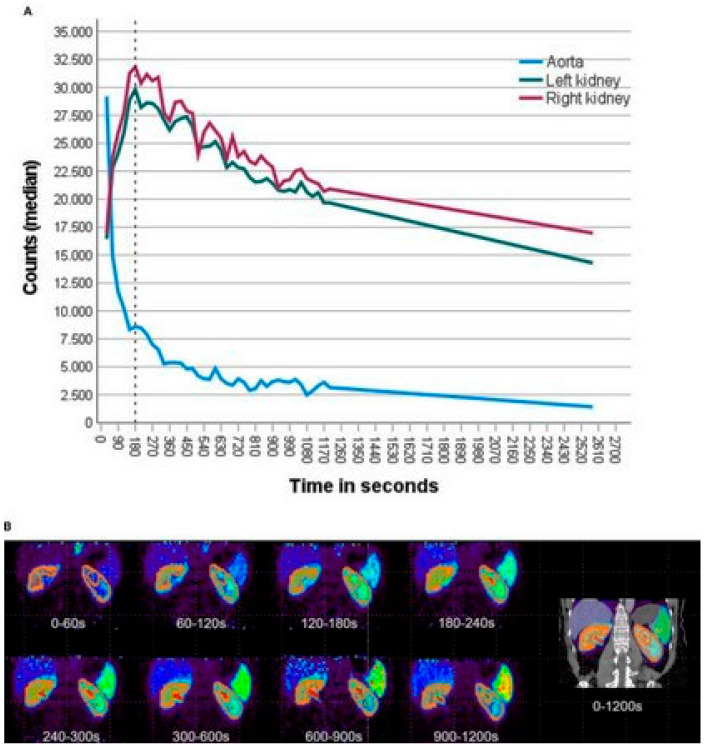
Dynamic SSR-PET imaging in a treatment-naïve NET patient with normal renal function revealed that the ^68^Ga-ha DOTATATE kinetics (**A**) renal parenchymal tracer concentration peaked at 180 s post-injection, followed by a gradual decline until 43 min. The blood pool activity (aorta) decreased exponentially. (**B**) Semiautomatic SUV-based segmentation (SUV 5–15 isocontour) effectively differentiated renal parenchyma from background/urine in static PET images, with clear temporal and spatial tracer distribution visualization. This figure is adapted from reference [23], available under a Creative Commons Attribution (CC BY 4.0) license.

Weissinger et al. [23] conducted a retrospective study to investigate the potential of using routine static ^68^Ga-ha DOTATATE PET/CT scans for non-invasive estimation of split renal function. This study aimed to determine if data from standard somatostatin receptor (SSR) PET/CT, commonly performed before peptide receptor radionuclide therapy (PRRT) for neuroendocrine tumors, could provide accurate renal function assessments, thus avoiding additional scintigraphy. This retrospective diagnostic accuracy study (n = 25 patients with metastatic neuroendocrine tumors) evaluated the feasibility of estimating GFR using static ^68^Ga-ha DOTATATE PET/CT scans acquired at 32 ± 0.5 min post-injection. GFR was calculated via the CKD-EPI formula (serum creatinine), with a mean baseline GFR of 79.4 ± 20.9 mL/min/1.73 m^2^ (range: CKD Stage 2–3b). Key PET-derived metrics included SUVmean of renal parenchyma, which showed a moderate negative correlation with GFR (r = −0.555, *p* < 0.001), and the novel Accumulation Index (ACI), defined as ^68^Ga-ha DOTATATE-avid renal parenchyma volume divided by SUVmean. ACI demonstrated a moderate positive correlation with GFR (r = 0.461, *p* < 0.05). Total Kidney Accumulation (TKA), however, showed no significant correlation (*p* = 0.135). The mean interval between PET and serum-based GFR measurement was 15.9 ± 40.6 days. Limitations included the small sample size, retrospective design, reliance on indirect GFR estimation (CKD-EPI), and lack of comparison to gold-standard GFR methods (e.g., inulin clearance). The study concluded that static ^68^Ga-SSR-PET/CT scans at 32 min p.i. may enable GFR estimation using ACI, though validation in larger cohorts and standardization of protocols is needed. This highlights PET’s potential as a non-invasive tool for renal function assessment in patients undergoing PRRT, with SUVmean and ACI as promising biomarkers [23] (Figure 5).

The inconsistent correlations between renal PET uptake and eGFR for different somatostatin receptor tracers could be attributed to their distinct molecular properties. ^68^Ga-DOTATATE exhibits high selectivity for the SSTR2 subtype, abundantly expressed in renal tubules, leading to significant tubular retention. Reflecting this, studies using this tracer have reported a negative correlation between renal SUVmean and eGFR [23]. In contrast, ^68^Ga-DOTATOC has a broader receptor affinity profile, resulting in different renal kinetics and, correspondingly, a reported lack of significant correlation with GFR in the reviewed literature [22]. This fundamental pharmacokinetic divergence confirms that quantitative renal uptake data are not interchangeable between SSR ligands.

#### 4.1.3. ^68^Ga-PSMA-11

**Figure 6:**
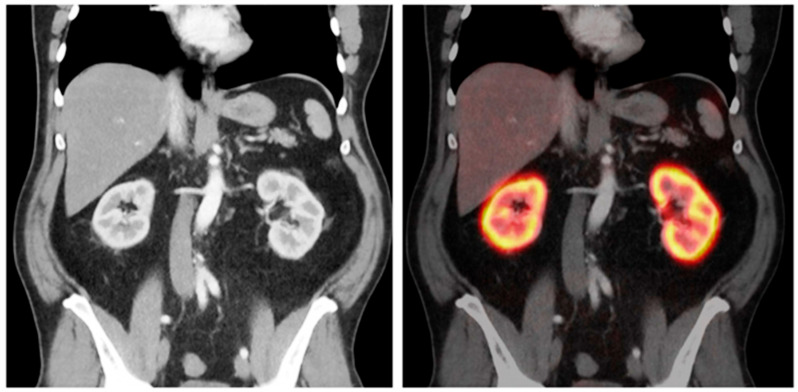
Illustration of renal uptake of a PSMA-targeted radiotracer on ^68^Ga-PSMA-11 PET/CT. (**Left**) Arterial-phase contrast-enhanced CT demonstrating clear differentiation between renal cortex and medulla. (**Right**) Fused PET/CT image showing prominent ^68^Ga-PSMA-11 accumulation within the renal cortex, with minimal uptake in surrounding abdominal organs. This figure is adapted from reference [25], available under a Creative Commons Attribution (CC BY 4.0) license.

**Figure 7:**
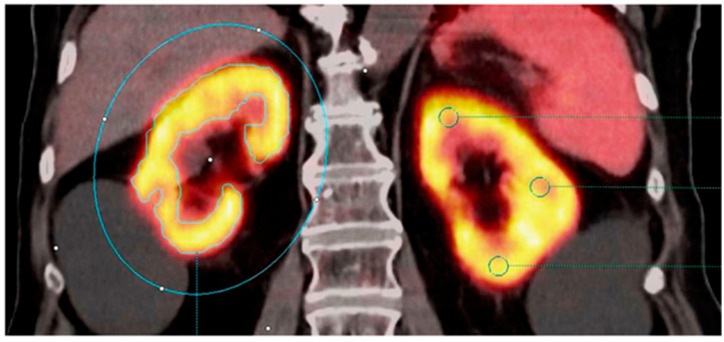
Quantification of renal ^68^Ga-PSMA-11 uptake on frontal PET/CT. Right kidney (left side of image): Whole-kidney VOI with 30% isocontour (blue) for SUV and volume measurements. Left kidney: Three 0.5 cm VOIs (green) for manual SUVmean assessment. Renal cysts without tracer uptake were excluded. This figure is adapted from reference [25], available under a Creative Commons Attribution (CC BY 4.0) license.

Gühne et al. conducted a retrospective study to evaluate the relationship between renal uptake of ^68^Ga-PSMA-11 in PET/CT imaging and kidney function, as assessed by eGFR. The study aimed to determine if ^68^Ga-PSMA-11 uptake could serve as an indicator of renal function, given the radiopharmaceutical’s prominent renal cortical uptake. Analyzing data from 103 male patients, the researchers measured renal uptake using various methods, including manual and computer-assisted contouring, and correlated these measurements with eGFR calculated via the CKD-EPI equation. The study revealed that while renal SUVmax, SUVpeak, and SUVmean did not correlate with eGFR, the molecular volume of the renal cortex showed a moderate correlation. Furthermore, the study found that a contouring threshold of 30% of SUVmax was optimal for quantifying renal cortex volume and total renal uptake. The authors concluded that ^68^Ga-PSMA-11 renal uptake intensity does not predict eGFR, but PET/CT imaging can quantify the functional renal cortex volume. This suggests that while ^68^Ga-PSMA-11 PET/CT provides valuable information about renal cortical volume, it is not a reliable tool for directly assessing overall kidney function as reflected by eGFR [25] (Figure 6 and Figure 7).

#### 4.1.4. ^68^Ga-FAPI

In addition to its established role in oncology, ^68^Ga-Fibroblast Activation Protein Inhibitor (FAPI) PET/CT has shown significant promise as a diagnostic tool in nononcological conditions. One such example is its application in assessing renal fibrosis in patients with chronic kidney disease (CKD). The novel PET radiotracer ^68^Ga-FAPI-04 targets fibroblast activation protein (FAP), overexpressed in fibrotic tissues, and holds promise for imaging renal fibrosis, a key contributor to CKD progression and declining GFR. Current diagnostic methods, such as renal biopsy, are invasive and have limitations in assessing the entire kidney. In this prospective study, 13 patients with histopathologically confirmed renal fibrosis underwent ^68^Ga-FAPI-04 PET/CT, with results compared to biopsy findings and immunohistochemical FAP expression. PET imaging demonstrated increased radiotracer uptake in affected renal tissue, correlating with fibrosis severity. The SUVmax increased with fibrosis grade (mild: 3.92 ± 1.50, moderate: 5.98 ± 1.6, severe: 7.67 ± 2.23). Furthermore, serum creatinine levels and GFR changes corresponded to fibrosis severity, supporting the clinical relevance of PET-derived parameters. Given its high sensitivity for detecting fibrosis non-invasively, ^68^Ga-FAPI-04 PET/CT provides a novel approach to renal fibrosis imaging, enabling early detection, disease monitoring, and therapy planning. These findings indicate that FAPI-based PET may complement, or in some cases reduce, the reliance on invasive renal biopsy, offering a promising tool for assessing CKD progression and fibrotic burden [19].

**Figure 8:**
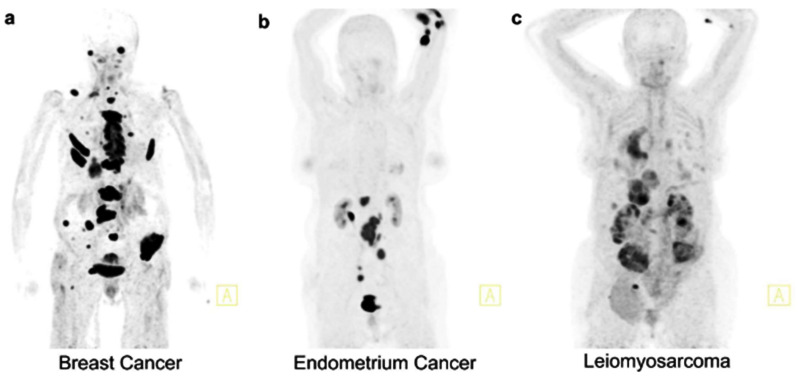
Comparison of 3 patients with different creatinine-levels undergoing a ^68^Ga-FAPI PET/CT scan. Creatinine levels are (**a**) 78.68 μmol/L, (**b**) 108.73 μmol/L, and (**c**) 223.66 μmol/L. The tracer uptake of the kidney parenchyma visually correlates with the increased creatinine levels with a clear renal uptake in the leiomyosarcoma patient and no tracer uptake in the kidneys of the breast cancer patient. PET/CT scans were performed 1 h p.i. indicated by the specified tumor diagnosis. A indicates the anterior plane in the figure. This figure is adapted from reference [24], available under a Creative Commons Attribution (CC BY 4.0) license.

**Figure 9:**
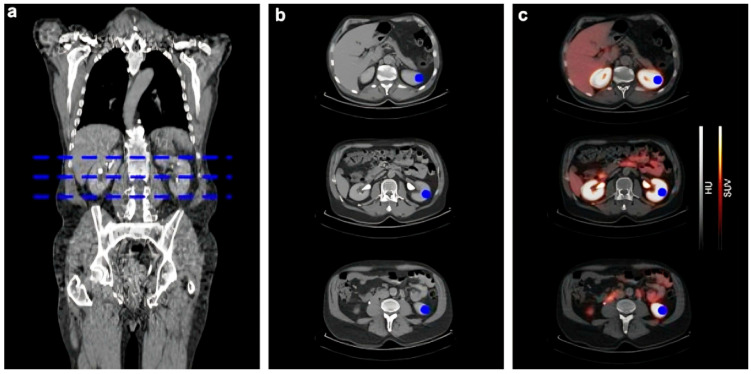
Illustration of renal tracer uptake measurement in a patient undergoing PSMA PET/CT. CT-based regions of interest (ROIs) are delineated in three orthogonal planes of the kidney (blue areas). ROI placement was performed identically for all three evaluated radiotracers ^68^Ga-FAPI-04, ^68^Ga-DOTATOC, or ^68^Ga-PSMA. Renal uptake was quantified using SUVmax and SUVmean at the defined locations. (**a**) Whole-body CT with blue dashed lines indicating the levels of three axial sections. (**b**) Corresponding CT slices showing manually drawn ROIs (blue). (**c**) Fused PET/CT images with ROIs superimposed (blue). This figure is adapted from reference [24], available under a Creative Commons Attribution (CC BY 4.0) license.

Another interesting study [24] that retrospectively evaluated the correlation between kidney function, measured by GFR, and renal uptake of three PET radiotracers, ^68^Ga-FAPI, ^68^Ga-PSMA, and ^68^Ga-DOTATOC, in 81 patients with varying stages of CKD. It demonstrated a strong negative correlation between renal SUVmax/SUVmean of ^68^Ga-FAPI and GFR (R^2^ = 0.57–0.66), indicating that higher tracer accumulation is associated with poorer kidney function and likely reflects active kidney fibrosis rather than passive retention or nonspecific tubular binding, Figure 8. In contrast, no significant correlation was found between GFR and renal uptake of ^68^Ga-DOTATOC or ^68^Ga-PSMA, although ^68^Ga-PSMA exhibited increased nonspecific background uptake with declining GFR (Figure 9).

### 4.2. ^18^F-Labelled Radiotracers

#### 4.2.1. Dynamic ^18^F-FDG-PET/MRI

**Figure 10:**
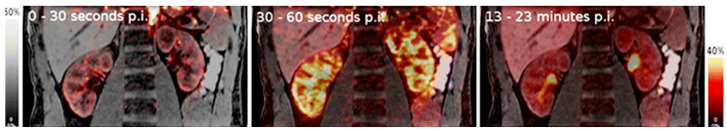
Adopted under CC BY 4.0 from [17] demonstrating the distribution of FDG in certain periods post injection (p.i.) obtained from a dynamic renal PET/MRI scan fusion image.

A prospective study by Geist et al. (2018) [17] investigates the feasibility of using ^18^F-FDG, a widely available PET radiotracer, to assess GFR and effective renal plasma flow (ERPF) in 24 patients via dynamic PET/MRI in healthy subjects. The authors demonstrated that ^18^F-FDG kinetics, analyzed using Patlak graphical methods and time–activity curves (TACs) guided by MRI-derived anatomical regions, strongly correlated with reference GFR (CKD-EPI formula: r = 0.88) and ERPF (^99m^Tc-MAG3 clearance: r = 0.82). However, reliance on indirect reference methods (e.g., serum creatinine-based GFR) and the exclusion of patients with renal pathology limit generalizability [17] (Figure 10).

#### 4.2.2. ^2^[^18^F]-FDS (2-Deoxy-2-^18^F-Fluoro-D-Sorbitol)

The novel PET radiotracer ^2^[^18^F]-FDS (2-deoxy-2-^18^F-fluoro-D-sorbitol) has demonstrated promising renal imaging properties due to its favorable kinetics and similarity to inulin clearance. This proof-of-concept a pilot study by Werner et al. (2019) [22] introduces ^18^F-FDS, a PET radiotracer derived from ^18^F-FDG, as a promising tool for functional renal imaging and GFR assessment. In two volunteers, dynamic PET/CT demonstrated its favorable renal kinetics., with cortical delineation reflecting blood flow, parenchymal transit, and urinary excretion. The tracer’s structural similarity to inulin is notable. ^18^F-FDS leverages existing ^18^F-FDG infrastructure, enhancing accessibility for clinical use, while its lower positron energy reduces radiation exposure, a critical advantage for pediatric applications. However, the study’s small sample size (n = 2) and lack of direct GFR correlation (e.g., against inulin or iohexol clearance) underscore the need for larger validation trials [22].

### 4.3. Other Tracers

#### 4.3.1. ^82^Rb-PET

While utilizing ^82^Rb-PET is mainly to assess renal blood flow (RBF) and renal vascular resistance (RVR), it does not directly measure GFR using PET radiotracers. Instead, it evaluates the correlation between RBF/RVR and eGFR (a serum-based estimation of GFR). A recent study evaluated the use of ^82^Rb-PET for assessing renal blood flow (RBF) and renal vascular resistance (RVR) in patients with varying renal function. The study included 51 patients, of whom 35 had normal renal function (eGFR ≥ 60 mL/min/1.73 m^2^) and 16 had abnormal renal function (eGFR < 60 mL/min/1.73 m^2^). Dynamic ^82^Rb-PET imaging with a one-compartment model demonstrated that RBF was significantly lower in the abnormal renal function group (median 173 vs. 443 mL/min/100 g, *p* = 0.022), while RVR was significantly higher (median 49.6 vs. 19.1 mmHg·min·g/mL, *p* = 0.0011). There was a moderate correlation between RBF and eGFR (r = 0.62, *p* < 0.0001) and an inverse correlation between RVR and eGFR (r = −0.59, *p* < 0.0001), suggesting that ^82^Rb-PET may provide a quantitative, noninvasive approach for assessing renal perfusion changes in impaired kidney function [26].

#### 4.3.2. ^64^Cu-ATSM PET/MRI

Renal perfusion serves as an indirect but essential indicator of GFR, reflecting the kidney’s ability to maintain adequate blood flow for filtration. In 2023, a feasibility study (n = 15: 10 CKD patients, 5 healthy controls [HCs]) evaluated renal blood flow (RBF) using ^64^Cu-ATSM PET/MRI, a novel quantitative approach, and validated it against arterial spin labeling MRI (ASL-MRI) and estimated RBF (eRBF) derived from clinical parameters. Participants underwent simultaneous PET/MRI imaging after ^64^Cu-ATSM injection (300–400 MBq), with dynamic PET data analyzed using a single-tissue compartment model and image-derived input function (IDIF) to generate PET-RBF. ASL-MRI provided reference RBF values, while eRBF was calculated from eGFR (serum creatinine/cystatin C), hematocrit, and filtration fraction. PET-RBF correlated strongly with eRBF (r = 0.893, *p* < 0.001) and ASL-RBF (r = 0.849, *p* < 0.001), with significant group differences in RBF (CKD vs. HCs: PET, 124 ± 22 vs. 151 ± 20 mL/min/100 g; ASL-MRI, 125 ± 30 vs. 172 ± 38 mL/min/100 g; *p* < 0.05). The study highlights ^64^Cu-ATSM PET/MRI as the first validated PET-based method for spatially resolved RBF quantification, demonstrating high concordance with ASL-MRI and clinical eRBF. Limitations include a small sample size (n = 15) and breath-holding artifacts in ASL-MRI. This work establishes ^64^Cu-ATSM PET/MRI as a promising dual-modality tool for CKD assessment, enabling precise, separate renal function evaluation with superior resolution to conventional nuclear methods [18].

## 5. Discussion

This systematic review highlights the emerging role of PET in renal functional imaging, particularly for GFR assessment and renal blood flow. Among the evaluated tracers, ^68^Ga-EDTA demonstrated the strongest validation against gold-standard reference methods such as ^51^Cr-EDTA, confirming its potential as a robust tool for quantitative renal imaging [21]. Its negligible protein binding and favorable clearance kinetics confer clear advantages over alternatives such as ^68^Ga-DTPA, which suffers from significant protein binding and consequent GFR underestimation [10]. These pharmacokinetic properties, combined with PET’s inherent strengths in high spatial resolution, dynamic imaging, and accurate quantification, position ^68^Ga-EDTA as a leading candidate for clinical translation.

Stable metal chelates such as EDTA and DTPA, which are exclusively cleared by glomerular filtration, have been radiolabeled with various isotopes for imaging and quantitatively assessing GFR. Although historically ^68^Ga-EDTA PET was used before ^99m^Tc-DTPA in renal imaging, its use declined with the rise of gamma camera technology, which was not well-suited for detecting positron decay. However, the resurgence of PET technology and broader availability of ^68^Ga have renewed interest in this tracer, which offers accurate, noninvasive, and quantitative evaluation of renal function. Modern camera-based clearance methods further enhance practicality by estimating GFR directly from renal tracer kinetics without the need for blood or urine sampling [28].

Before 2023, clinicians lacked access to cold kit for the preparation of ^68^Ga-EDTA for immediate diagnostic use, hindering its adoption in routine practice. To address this gap, Skulska et al. developed and validated a GMP-compliant (Good Manufacturing Practice-compliant), kit-based synthesis of ^68^Ga-EDTA, bridging the gap between preclinical findings and clinical application. Their study detailed the design, production, quality control, and stability of the kit, emphasizing a standardized radiolabeling procedure using a ^68^Ge/^68^Ga generator and automated synthesis. The validated formulation, composed of disodium EDTA dihydrate and a tailored buffer system, mitigated ^68^Ga colloid formation, ensuring reproducible labeling and high radionuclide purity. By leveraging ^68^Ga’s favorable decay characteristics, including high positron-emission fraction and a suitable half-life [29].

Despite PET’s widespread use in oncology, neurology, and cardiology, nephrology has yet to benefit fully from its diagnostic precision. Clinical studies remain limited in number and scale, with most focusing on proof-of-concept validation rather than large-scale clinical application. Importantly, while ^68^Ga-EDTA offers strong accuracy in normal and impaired renal function, its tendency to underestimate hyperfiltration (>150 mL/min) suggests it may be less suited for living kidney donor evaluation, though highly relevant in CKD monitoring and transplant follow-up [30].

PET also offers advantages over conventional nuclear medicine modalities. Planar imaging lacks anatomical detail, and SPECT suffers from limited temporal resolution and challenges with dynamic acquisitions. PET, in contrast, enables precise time–activity curve generation, accurate split renal function measurement, with lower radiation exposure (<2 mSv for ^68^Ga-EDTA PET), a key advantage for pediatric patients needing repeated evaluations [1].

Beyond ^68^Ga-EDTA, several novel tracers hold promise. Fluorine-18 agents such as ^18^F-FDS mimic inulin clearance and show potential as true GFR tracers with low protein binding and high stability, though clinical validation is minimal [22]. Hybrid PET/MRI enables simultaneous functional and anatomical imaging, with 15 min scans showing accuracy comparable to 30 min protocols (ICC 0.96–0.99), streamlining workflow for routine clinical use [17,18].

Clinical adoption of PET-based renal assessment depends on practical factors. Availability is foremost; while ^18^F-FDG benefits from widespread distribution, ^68^Ga-based tracers depend on local ^68^Ge/^68^Ga generator access, and ^64^Cu-ATSM necessitates a cyclotron. Workflow is influenced by scan duration; dynamic imaging (20–30 min) enables precise GFR kinetics, while static scans offer faster but less quantitative surrogates. Although costs exceed those of serum tests or scintigraphy, PET provides superior accuracy, 3D localization, and multiparametric data. Selection should be guided by the clinical question: filtered agents (^68^Ga-EDTA, ^18^F-FDS) for GFR measurement, perfusion tracers (^82^Rb, ^64^Cu-ATSM) for blood flow, and oncologic tracers (^68^Ga-PSMA-11, ^68^Ga-DOTATATE) for opportunistic renal evaluation during existing scans [31].

Preclinical studies provide important mechanistic validation, but their clinical translation remains limited. Tracers such as ^68^Ga-EDTA, ^18^F-FDS, and ^64^Cu-based compounds have consistently demonstrated favorable kinetics, sensitivity to early dysfunction, and superior spatiotemporal resolution compared to conventional methods in animal models. However, interspecies differences, protocol heterogeneity, and reliance on small single-center studies limit generalizability. Robust clinical validation across diverse patient populations, including CKD, transplant recipients, and pediatric cohorts, remains essential before these tracers can be integrated into practice.

The summarized table highlights the diverse pharmacokinetic profiles and clinical utilities of current PET radiotracers for GFR measurement, emphasizing their distinct advantages such as superior spatial–temporal resolution, non-invasiveness, and potential for simultaneous anatomical-functional imaging, while also acknowledging limitations including variable validation levels and tracer-specific accuracy constraints; notably, no direct comparisons between novel PET tracers and the established reference method of plasma clearance of ^99^ᵐTc-DTPA have been reported, underscoring a gap for future head-to-head studies (Table 2).

An ongoing study (NCT06973798) is evaluating the utility of ^68^Ga-HBED-CC-DiAsp PET/CT for both quantifying GFR and predicting postoperative residual renal function in patients undergoing partial nephrectomy. The study compares PET-derived measurements with conventional methods, including the Gates method and dual plasma sampling with ^99m^Tc-DTPA, using intra-class correlation, concordance correlation, and Bland–Altman analyses to assess agreement. Although results are not yet available, this trial may provide important insights into the role of novel PET radiotracers in perioperative renal function assessment [32].

A key limitation across most included studies is their reliance on eGFR, derived from serum creatinine, as the reference standard. While eGFR is a vital clinical screening tool, its use for validating a novel measurement method is fundamentally flawed. eGFR is itself an imperfect estimate, influenced by non-renal factors such as muscle mass and diet. Validating a new technique against another estimate precludes a true assessment of accuracy and creates a circular argument. This methodological weakness underscores that future studies must validate PET-based GFR against direct measurement gold standards, such as inulin, iohexol, or ^51^Cr-EDTA plasma clearance.

Moreover, despite its quantitative advantages, the broader clinical adoption of PET-based renal functional imaging remains constrained by the limited availability of cyclotron-dependent tracers particularly short-lived compounds such as ^15^O-water and ^11^C-labeled agents [31]. Radiochemistry for synthesizing inulin analogs, while scientifically promising, remains technically demanding and lacks standardized protocols. and a lack of harmonized kinetic modeling protocols. Furthermore, its higher operational cost compared to SPECT remains a significant barrier to widespread adoption.

Future work must therefore prioritize multicenter prospective trials; standardized imaging protocols; and tracer-specific calibration to address known limitations, such as the underestimation of hyperfiltration. Advances in PET technology, including time-of-flight imaging and improved reconstruction algorithms, may further enhance diagnostic accuracy while reducing dose. If validated in larger populations, PET-based GFR measurement could complement or even replace existing plasma clearance methods, offering clinicians a quantitative, reproducible, and low-dose alternative for renal functional assessment.

## 6. Conclusions

PET radiotracers, particularly ^68^Ga-EDTA, demonstrate strong potential for accurate, non-invasive measurement of GFR with distinct advantages over conventional nuclear medicine techniques. Their superior spatial and temporal resolution, combined with three-dimensional quantification and ability for split renal function assessment, enable comprehensive renal evaluation beyond plasma clearance methods. While tracers like ^18^F-FDS and 64Cu compounds show promising kinetics paralleling gold-standard markers such as inulin, clinical validation remains limited.

The current evidence, drawn from mostly small-scale and proof-of-concept studies, highlights PET’s promise but also underscores significant limitations including methodological heterogeneity, retrospective designs, and underrepresentation of diverse patient populations. Notably, PET radiotracers tend to underestimate GFR in hyperfiltration states, signaling the need for tracer-specific calibration.

Future research should focus on prospective, multicenter trials with standardized imaging protocols and direct comparisons to established gold standards to confirm diagnostic accuracy and clinical utility. Advances in PET technology and simplified acquisition protocols may facilitate broader clinical adoption. Overall, PET-based GFR measurement represents a transformative step toward precise, quantitative renal functional imaging with potential to improve management in chronic kidney disease, transplantation, and pediatric patients.

## Figures and Tables

**Table 1:** Characteristics of the included studies.

Authors	year	Month	Location	Type of Study	Sample Size	Reference Standard Used in GFR Measurement	PET Radiotracer
Hofman et al. [21]	2015	3	Australia	Original/Prospective	25	Plasma clearance of ^51^Cr-EDTA (^68^Ga-EDTA was the experimental tracer).	^68^Ga-EDTA
Kaewput et al. [27]	2016	12	Thailand	Original/Retrospective Observational	32	Estimated GFR (eGFR) Cockcroft-Gault formula (adjusted for body surface area), derived from serum creatinine.	^68^Ga-DOTANOC
Geist et al. [17]	2018	5	Austria	Original/Prospective	24	eGFR using the CKD-EPI formula, derived from serum creatinine.	^18^F-FDG
Werner et al. [22]	2019	5	Germany	Human Pilot/First-in-Human	2	No reference GFR standard reported (feasibility study)	^18^F-FDS
Zhou et al. [19]	2021	2	China	Original/Diagnostic Accuracy Prospective	13	eGFR (CKD-EPI), derived from serum creatinine. Renal biopsy was the reference for fibrosis, not GFR.	^68^Ga-FAPI-04
Kersting et al. [20]	2022	8	Germany	Original/Diagnostic Accuracy Prospective	12	eGFR (CKD-EPI), derived from serum creatinine.	^68^Ga-DOTA
Conen et al. [24]	2022	8	Germany	Original/Retrospective	81	Tubular extraction rate (TER) from ^99^ᵐTc-MAG3 scintigraphy and eGFR (CKD-EPI).	^68^Ga-FAPI, ^68^Ga-PSMA and ^68^Ga-DOTATOC
Weissinger et al. [23]	2023	6	Germany	Original/Diagnostic Accuracy Retrospective	25	Reference standard tubular extraction rate (TER-MAG) from ^99m^Tc-MAG3 scintigraphy and GFR CKD-EPI formula from serum creatinine.	^68^Ga-ha DOTATATE
Nishikawa et al. [18]	2023	1	Japan	Original/Prospective	15	Estimated Renal Blood Flow (eRBF) calculated from eGFR (CKD-EPI formula).	^64^Cu-ATSM
Guhne et al. [25]	2024	3	Germany	Original/Retrospective Observational	103	eRBF calculated from eGFR (CKD-EPI formula).	^68^Ga-PSMA-11
Bibeau-Delisle et al. [26]	2024	11	Canada	Original/Retrospective Observational	51	eGFR (CKD-EPI), derived from serum creatinine.	^82^Rb-RbCl

**Table 2:** Overview of PET radiotracers used in GFR measurement. The table summarizes key radiotracers, their isotopic labels, pharmacokinetic and functional characteristics, main clinical or research findings on GFR estimation or renal function assessment, advantages including imaging quality or safety, and limitations or practical considerations.

Radiotracer	Radioisotope Label	Key Characteristics	Main Clinical/Research Findings	Advantages	Limitations/Notes
^68^Ga-EDTA	Gallium-68	Stable metal chelate, low protein binding, exclusive glomerular filtration	Strong correlation with reference ^51^Cr-EDTA clearance (r = 0.94), suitable for split renal function assessment	High spatial/temporal resolution, 3D quantification, low radiation dose	Underestimates GFR >150 mL/min, scarce large prospective validation
^68^Ga-DOTANOC	Gallium-68	Somatostatin receptor targeting	Weak correlation with eGFR; uptake increased post peptide receptor radionuclide therapy (PRRT)	Potential early biomarker for renal toxicity post-PRRT	Limited correlation with GFR, retrospective studies
^18^F-FDG	Fluorine-18	Widely available PET tracer, not renal specific	Dynamic PET/MRI showed correlation with GFR (r = 0.88), effective renal plasma flow estimation	Dual-purpose oncologic and renal function imaging	Indirect reference method, limited in renal pathology
^18^F-FDS	Fluorine-18	Structural similarity to inulin, low protein binding	Pilot study: favorable renal kinetics, potential GFR tracer, sorbitol-to-inulin clearance 1.01	Compatible with existing PET infrastructure, lower radiation	Very limited clinical data, small sample size
^68^Ga-PSMA-11	Gallium-68	Prostate-specific membrane antigen targeting	Moderate correlation of renal cortex volume with eGFR; SUV measures not correlating	Quantifies renal cortex volume with good anatomical detail	Not reliable for direct GFR measurement
^68^Ga-FAPI-04	Gallium-68	Targets fibroblast activation protein in fibrosis	Uptake correlates with renal fibrosis severity, SUVmax increases with fibrosis grade	Non-invasive fibrosis imaging, potential complement to functional GFR	Needs validation against biopsy, limited clinical data
^64^Cu-ATSM	Copper-64	PET/MRI for renal blood flow quantification	Strong correlation with ASL-MRI and estimated RBF, differentiates CKD from healthy controls	First validated PET method for spatial RBF; dual modality	Small sample size, still indirect for GFR
^68^Ga-DOTA	Gallium-68	Small-molecule filtration agent used in dynamic imaging.	Good correlation with serum creatinine-derived GFR; shorter scan protocols feasible	Enables dynamic renal function and anatomical imaging	Reliant on surrogate reference GFR methods
^68^Ga-ha DOTATATE	Gallium-68	Somatostatin receptor targeting	Moderate correlation of novel PET metrics with GFR; potential for split renal function assessment	Possible non-invasive GFR estimation from routine PET scans	Retrospective, indirect GFR references
^82^Rb-RbCl	Rubidium-82	Primary use in myocardial perfusion imaging; investigational for renal blood flow assessment	Correlation of RBF and eGFR demonstrated	Non-invasive renal perfusion assessment	Does not directly measure GFR

## Data Availability

No new data were created or analyzed in this study. Data sharing is not applicable to this article.

## Supplementary Materials

The following supporting information can be downloaded at: https://www.mdpi.com/article/10.3390/diagnostics15243209/s1, Table S1: PRISMA 2020 Checklist.

## Abbreviations

The following abbreviations are used in this manuscript:

GFR	Glomerular Filtration Rate
SUV	Standardized Uptake Value
SUVmax	Maximum Standardized Uptake Value
SUVmean	Mean Standardized Uptake Value
TAC	Time–Activity Curve
TER	Tubular Extraction Rate
TKA	Total Kidney Accumulation
TLG	Total Lesion Glycolysis
TLGSUL	Total Lesion Glycolysis corrected for Lean Body Mass (SUL)
VOI	Volume of Interest
^18^F-FDG	2-deoxy-2-[^18^F]fluoro-D-glucose
^18^F-FDS	2-deoxy-2-[^18^F]fluoro-D-sorbitol
^51^Cr-EDTA	Chromium-51 ethylenediaminetetraacetic acid
^64^Cu-ATSM	Copper-64 diacetyl-bis(N4-methylthiosemicarbazone)
^68^Ga	Gallium-68
^68^Ga-DOTA	Gallium-68 1,4,7,10-tetraazacyclododecane-1,4,7,10-tetraacetic acid
^68^Ga-DOTANOC	Gallium-68 1,4,7,10-tetraazacyclododecane-1,4,7,10-tetraacetic acid-1-Nal3-octreotide
^68^Ga-EDTA	Gallium-68 ethylenediaminetetraacetic acid
^68^Ga-FAPI	Gallium-68 Fibroblast Activation Protein Inhibitor
^68^Ga-ha DOTATATE	Gallium-68 1,4,7,10-tetraazacyclododecane-1,4,7,10-tetraacetic acid-Tyr3-octreotate
^68^Ga-PSMA-11	Gallium-68 prostate-specific membrane antigen-11
^82^Rb-RbCl	Rubidium-82 Rubidium Chloride
^90^Y	Yttrium-90
^99^ᵐTc-DTPA	Technetium-99m diethylenetriaminepentaacetic acid
^99^ᵐTc-MAG3	Technetium-99m mercaptoacetyltriglycine
ACI	Accumulation Index
ASL-MRI	Arterial Spin Labeling Magnetic Resonance Imaging
BSA	Body Surface Area
CKD	Chronic Kidney Disease
CKD-EPI	Chronic Kidney Disease Epidemiology Collaboration
CT	Computed Tomography
eGFR	Estimated Glomerular Filtration Rate
ERPF	Effective Renal Plasma Flow
FAP	Fibroblast Activation Protein
MRI	Magnetic Resonance Imaging
NET	Neuroendocrine Tumor
PET	Positron Emission Tomography
PCC	Pearson Correlation Coefficient
SPECT	Single Photon Emission Computed Tomography
SSR	Somatostatin Receptor
PRRT	Peptide Receptor Radionuclide Therapy
RBF	Renal Blood Flow
ASL-MRI	Arterial Spin Labeling MRI
